# A Quantitative Model Explains Single-Cell Dynamics of the Adaptive Response in *Escherichia coli*

**DOI:** 10.1016/j.bpj.2019.08.009

**Published:** 2019-08-15

**Authors:** Stephan Uphoff

**Affiliations:** 1 Department of Biochemistry, University of Oxford, Oxford, United Kingdom

## Abstract

DNA damage caused by alkylating chemicals induces an adaptive response in *Escherichia coli* that increases the tolerance of cells to further damage. Signaling of the response occurs through irreversible methylation of the Ada protein, which acts as a DNA repair protein and damage sensor. Methylated Ada induces its own gene expression through a positive feedback loop. However, random fluctuations in the abundance of Ada jeopardize the reliability of the induction signal. I developed a quantitative model to test how gene expression noise and feedback amplification affect the fidelity of the adaptive response. A remarkably simple model accurately reproduced experimental observations from single-cell measurements of gene expression dynamics in a microfluidic device. Stochastic simulations showed that delays in the adaptive response are a direct consequence of the very low number of Ada molecules present to signal DNA damage. For cells that have zero copies of Ada, response activation becomes a memoryless process that is dictated by an exponential waiting time distribution between basal Ada expression events. Experiments also confirmed the model prediction that the strength of the adaptive response drops with an increasing growth rate of cells.

## Significance

Gene expression noise can influence cell fates and diversify phenotypes in response to stress. For genotoxic stress, variable expression of DNA repair genes will modulate crucial genome maintenance mechanisms, which can affect an individual cell’s chance of survival or rate of mutagenesis. This study addresses the role of gene expression noise in the adaptation of *Escherichia coli* to DNA alkylation damage. A quantitative model of the gene regulatory circuit of the adaptive response along with stochastic simulations explained observations from single-cell microfluidics experiments across a broad range of conditions and perturbations. The model shows that stochastic expression of Ada—a DNA methyltransferase and transcriptional activator—is responsible for creating stark cellular heterogeneity in the adaptive response.

## Introduction

The accurate detection and repair of DNA damage is crucial for genome stability and cell survival. In addition to constitutively expressed repair pathways, cells employ DNA damage responses that activate DNA repair factors in the presence of DNA damage. The fidelity of the DNA repair system relies on a series of processes: sensing the presence of DNA damage or DNA damaging agents, inducing a DNA damage response, and correctly repairing lesions. Cells with genetic defects that impair the function of any of these processes show sensitivity to DNA damage, elevated mutation rates, and genome instability. However, even in fully repair-proficient strains, the accuracy of the DNA repair system is fundamentally limited by the stochastic nature of the molecular interactions involved (1, 2): For example, proteins that signal or repair DNA damage perform a random target search and therefore have a finite chance of overlooking lesions (3, 4, 5, 6, 7). Furthermore, the repair process itself can be error prone and cause mutations, loss, or rearrangements of genetic material (8, 9, 10, 11, 12). Traditionally, research has focused on genetic defects and such “intrinsic errors” in DNA repair–i.e., errors that are inherent to the repair mechanism and thus occur with the same probability in all cells of a population.

By comparison, less attention has been given to “extrinsic variation” in the DNA repair system–i.e., fluctuations in protein abundances that may affect the repair capacity of individual cells. In fact, gene expression noise is ubiquitous (13), difficult for cells to suppress (14), and is the source of phenotypic heterogeneity that is widely observed in isogenic cell populations (15). Feedback gene regulation can establish bimodal distributions so that subpopulations of cells maintain distinct states of gene expression for long times. Whereas many biological processes are robust to a certain level of noise, even transient variation in the capacity of a cell to repair DNA damage can have severe and potentially irreversible consequences (16, 17, 18). For instance, cells that transiently express too little of a damage sensor protein may be unable to signal DNA damage efficiently, leading to mutations or cell death. But there are also evolutionary benefits to heterogeneity and occasional errors in DNA repair processes when cells are facing selective pressures (19, 20, 21). Because noise in DNA repair may tilt the balance between genome maintenance and plasticity, it may play an important role in modulating the rate of adaptive evolution on a single-cell level. This point is of particular interest with regards to the role of phenotypic heterogeneity in the evolution of drug resistance in microbes and cancers (22, 23, 24).

The adaptive response to DNA alkylation damage in *Escherichia coli* is a case in which gene expression noise appears to cause significant cell-to-cell heterogeneity in DNA repair capacity (17, 18). Alkylating agents, such as methyl methanesulfonate (MMS), occur endogenously and in the environment. In cells, alkylated DNA lesions block DNA replication and transcription and can lead to mutations (10, 25). The adaptive response is regulated by the Ada protein, a DNA methyltransferase that directly repairs methylated phosphotriester, O^6^MeG, and O^4^MeT lesions by transferring the methyl groups from the DNA onto two cysteine residues on itself (26, 27, 28). These reactions are irreversible and turn the methylated Ada protein (meAda) into a transcriptional activator of the genes *ada*, *alkB*, *alkA*, and *aidB* that are involved in the repair or prevention of DNA alkylation damage (29). The response causes a positive feedback amplification of Ada expression that renders cells more tolerant to further damage. No demethylation reactions have been reported for meAda. Therefore, deactivation of the adaptive response is thought to occur via the dilution of meAda molecules due to cell growth, inhibition of transcriptional activation by competition with unmethylated Ada (30), and possibly proteolytic cleavage (31). Surprisingly, the timing of response activation varies drastically across genetically identical cells, even at saturating levels of DNA damage (18). The very low abundance of Ada before DNA damage treatment appears to be the cause of this variation. In particular, single-molecule imaging showed that stochastic Ada expression results in a subpopulation of cells that does not contain a single Ada protein and therefore cannot sense the presence of DNA alkylation damage. Without induction of the adaptive response, the insufficient repair capacity of these cells increases mutagenesis to a similar level as in mutant cells in which the *ada* gene has been deleted (17).

These surprising observations call for a quantitative model to pinpoint the noise source underlying the heterogeneity in the adaptive response. Quantitative models have been key to our current understanding of gene expression noise and its important functions in diverse biological processes (13, 32, 33, 34, 35), including DNA damage signaling and repair (16, 36, 37, 38, 39). Here, I capitalized on time-lapse microscopy data showing the adaptive response dyanamics in hundreds of single *E. coli* cells for a large range of damage conditions (18). The direct measurement of key observables and parameters allowed construction of a quantitative model of the core Ada regulation. The proposed model is remarkably simple yet accurately reproduces experimental observations—both the cell average as well as the stochastic behavior of single cells. The model also predicts cell responses after different experimental perturbations. No additional post hoc noise term was required in this model, but propagation of basic Poisson fluctuations alone was sufficient to explain the observed cell-to-cell variation in response activation. These results establish that intrinsic noise in the basal expression of the *ada* gene is solely responsible for the stochastic nature of the adaptive response. The model also predicts that the strength of the response should be inversely related to the growth rate of cells, which was confirmed in experiments.

## Materials and Methods

### Single-cell imaging of the adaptive response

The construction of the model was based on experimental data described in (18). Briefly, the adaptive response was monitored in live *E. coli* AB1157 cells carrying a functional fusion of Ada to the fast-maturing fluorescent protein mYPet (40) that is expressed from the endogenous chromosomal locus, thus maintaining native expression levels. Cells also expressed mKate2 constitutively to aid automated segmentation image analysis. Fluorescence time-lapse imaging was performed at 3-min time intervals on a Nikon Ti-E inverted fluorescence microscope (Nikon, Tokyo, Japan) equipped with a 60× NA 1.40 oil immersion objective and Hamamatsu Orca R2 CCD camera (Hamamatsu, Shizuoka, Japan). Single fluorescence snapshots were recorded on a similar Nikon Ti-E instrument equipped with a 100× NA 1.4 oil immersion objective and Photometrics CoolSNAP HQ CCD camera (Teledyne Photometrics, Tucson, AZ). Both instruments had an LED excitation source (Lumencor SpectraX; Lumencor, Beaverton, OR) and perfect focus system. Exposure times were 50 ms for mKate2 and 100 or 200 ms for Ada-mYPet (time lapse or snapshots). Cells were grown and imaged at 37°C in supplemented M9 minimal medium containing M9 salts (15 g/L KH_2_PO_4_, 64 g/L Na_2_HPO_4_, 2.5 g/L NaCl, and 5.0 g/L NH_4_Cl), 2 mM MgSO_4_, 0.1 mM CaCl_2_, 0.5 *μ*g/mL thiamine, minimal essential medium amino acids, 0.1 mg/mL L-proline, 0.85 mg/mL Pluronic F127 (added for microfluidics), and 0.2% glucose (or 0.2% glycerol as indicated in Fig. 5). Microfluidic single-cell imaging was performed using the “mother machine” microfluidic device (41). A constant flow of growth medium containing the DNA methylating agent MMS was applied at the times and concentrations indicated in the figures. For snapshots, shaking cultures were treated with MMS before imaging immobilized cells on agarose pads.

### Ada response model

**Figure 1:**
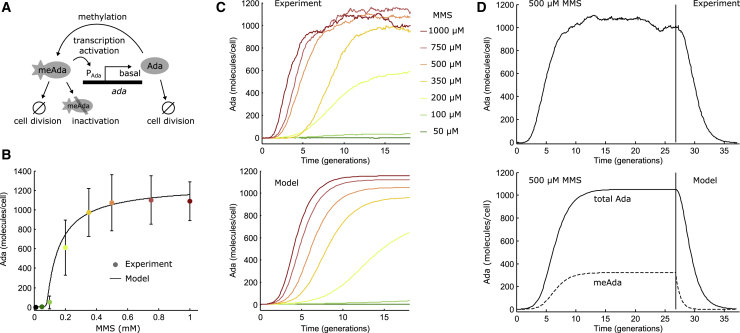
Deterministic model of the adaptive response. (*A*) Schematic of the model is shown; Ada is expressed at a low basal level in undamaged cells. MMS treatment creates DNA methylation damage that converts Ada to meAda. Transcription of ada is activated by meAda binding to the P_Ada_ promoter. Both Ada and meAda molecules are diluted because of cell growth and division. Additionally, meAda gets inactivated by degradation or autorepression. (*B*) Shown is the cell average steady-state expression of Ada after constant treatment with different doses of MMS for 20 cell generations (mean ± SD). The curve shows the analytical steady-state solution of the model for the total Ada abundance (Ada + meAda + inAda). (*C*) Shown is the cell average response when MMS was added at time 0 and Ada abundance was measured by time-lapse fluorescence microscopy. The model curves of total Ada abundance were generated by numerically solving the rate equations for different MMS concentrations. (*D*) Shown is the cell average response and response deactivation upon the addition and removal of 500 *μ*M MMS during time-lapse microscopy. The vertical line indicates the time of MMS removal. The numerical solution of the model shows total Ada and meAda abundances as solid and dashed lines, respectively. To see this figure in color, go online.

The structure of the model is based on previous genetic and biochemical characterization of the adaptive response (26, 27, 28). Key to the model is a positive feedback loop in which DNA damage-induced irreversible methylation of Ada creates meAda, which acts as a transcriptional activator for the *ada* gene. The chemical kinetics of the model can be described as a system of ordinary differential equations according to the diagram in Fig. 1 *A*:

(1) $$d/\mathrm{dt}\;\left[\mathrm{meAda}\right]=f_{1}\left(\left[\mathrm{MMS}\right]\right)\cdot \left[\mathrm{Ada}\right]–\left(\ln\left(2\right)\;+\rho \right)\cdot \left[\mathrm{meAda}\right],$$

(2) $$d/\mathrm{dt}\;\left[\mathrm{Ada}\right]=k_{basal}+f_{2}\left(\left[\mathrm{meAda}\right]\right)–\ln\left(2\right)\;\cdot \;\left[\mathrm{Ada}\right].$$

Self-methylation of Ada in the presence of DNA methylation damage generates meAda molecules with a rate proportional to the MMS concentration: *f*_*1*_([MMS]) = *k*_me_ ⋅ [MMS].

In the absence of DNA methylation damage, the *ada* gene is expressed at a constant basal rate *k*_basal_ from the P_Ada_ promoter. This rate is independent of the abundance of Ada or meAda. Transcription of the *ada* gene is induced to rate *k*_ind_ when meAda binds to the P_Ada_ promoter with an association rate *k*_on_ and dissociation rate *k*_*off*_ in a noncooperative manner (42):

$$P_{\mathrm{Ada}}+\mathrm{meAda}↔P_{\mathrm{Ada}}-\mathrm{meAda}.$$

In the deterministic model, Ada is produced according to the fraction of time that the P_Ada_ promoter is bound by meAda:

$$f_{2}\left(\left[\mathrm{meAda}\right]\right)=k_{\mathrm{ind}}\cdot \left[\mathrm{meAda}\right]/\left(k_{\mathrm{off}}/k_{\mathrm{on}}+\left[\mathrm{meAda}\right]\right),$$

where *k*_ind_ is the fully induced production rate at saturating amounts of meAda.

Production of Ada and meAda molecules is counteracted by dilution due to exponential cell growth. When time is expressed in units of generation times, the dilution rate is equal to ln (2) in the deterministic model. In addition to dilution, the model also includes loss of meAda at a constant rate *ρ*. This feature can account for proteolytic cleavage of Ada (31) or other mechanisms that can inhibit transcriptional activation by meAda (30). The equation governing the concentration of the inactivated Ada species is as follows:

(3) d/dt[inAda]=ρ⋅[meAda]–ln(2)⋅[inAda].

The model approximates protein expression as one reaction in which transcription and translation are described with a single production rate constant. This reduces the number of free parameters of the model and allows direct comparison of the experimental observables (i.e., Ada-mYPet proteins) with the outputs of the model. The total Ada level corresponding to the measured Ada-mYPet fluorescence is given by the sum of [Ada] + [meAda] + [inAda]. Bundling transcription and translation is valid when protein expression follows first-order kinetics with a single rate-limiting step. This is consistent with the complete lack of *ada* expression bursting in our experiments (18) and a short half-life and low translation efficiency of *ada* messenger RNAs (mRNAs) (43, 44).

### Steady-state solution

Setting Eqs. 1, 2, and 3 to zero gives the abundances of Ada and meAda at steady state. These can be expressed as the solution of a quadratic equation as follows:

$$\left[\mathrm{Ada}\right]=–b/\left(2a\right)+1/\left(2a\right)\cdot \surd \left(b^{2}–4ac\right),$$

with

$$a=k_{me}\cdot \left[\mathrm{MMS}\right]/\left(\ln\left(2\right)+\rho \right),$$

$$b=k_{\mathrm{off}}/k_{\mathrm{on}}–\left(k_{\mathrm{basal}}+k_{\mathrm{ind}}\right)\cdot k_{\mathrm{me}}\cdot \left[\mathrm{MMS}\right]/\left(\ln\left(2\right)+k_{\mathrm{me}}\cdot \left[\mathrm{MMS}\right]\cdot \left(\ln\left(2\right)+\rho \right)\right),$$

$$c=–k_{\mathrm{basal}}\cdot k_{\mathrm{off}}/k_{\mathrm{on}}/\left(\ln\left(2\right)+k_{me}\cdot \left[\mathrm{MMS}\right]\right),$$

$$\left[\mathrm{meAda}\right]=k_{\mathrm{me}}\cdot \left[\mathrm{MMS}\right]\;\cdot \;\left[\mathrm{Ada}\right]/\left(\ln\left(2\right)+\rho \right),$$

[inAda]=ρ/ln(2)⋅[meAda].

### Numerical solution

The time-dependent solution of the model equations was numerically obtained using the ode45 solver in MATLAB (The MathWorks, Natick, MA).

### Stochastic simulation

**Figure 2:**
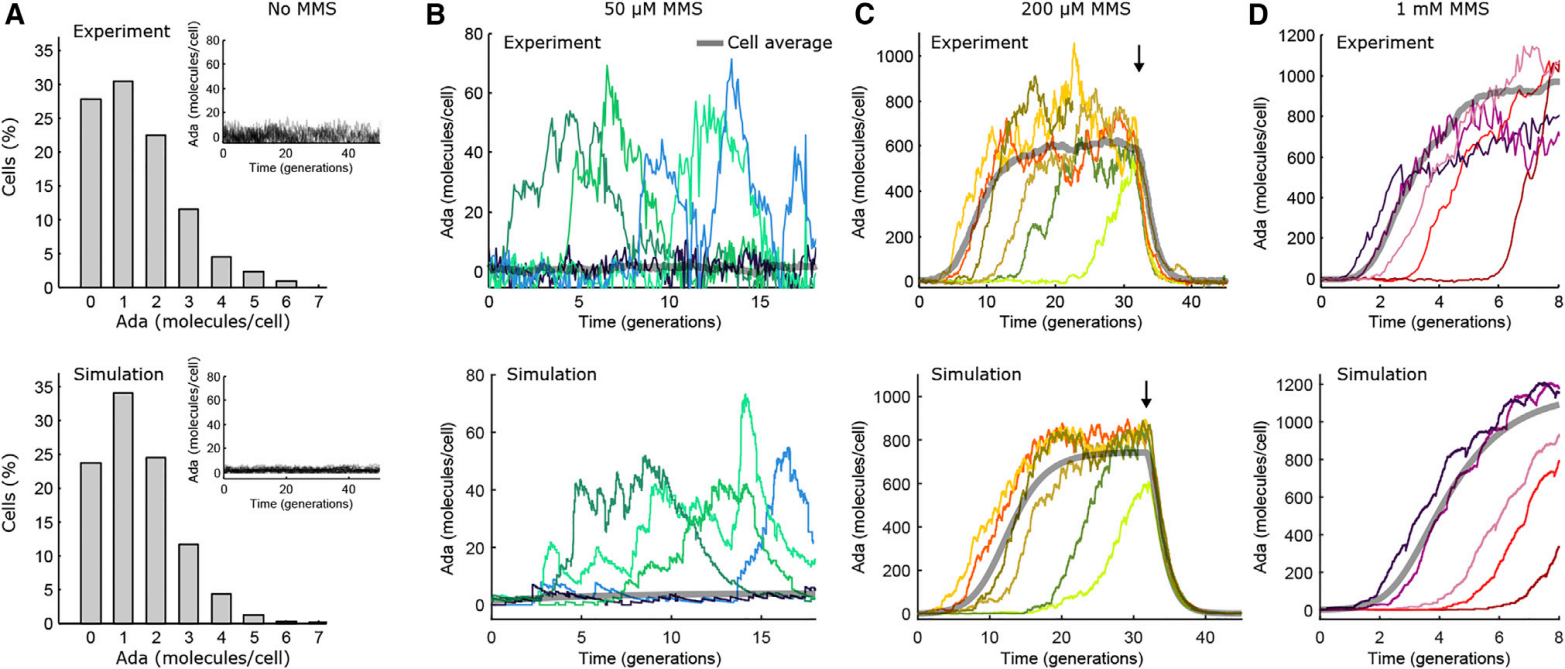
Stochastic dynamics of the adaptive response in single cells. (*A*) Distribution of the number of Ada molecules per cell without MMS treatment is shown. The experimental data (*top*) are from single-molecule counting experiments (18). Simulated data (*bottom*) were drawn from a Poisson distribution with a mean of one per cell. Inset: single-cell time traces of Ada expression without MMS treatment is shown. (*B*–*D*) Example time traces show random unsynchronized pulses of Ada expression with 50 *μ*M MMS treatment and stochastic activation of the Ada response with 200 *μ*M and 1 mM MMS treatment. Example time traces from time-lapse microscopy experiments (*top*) and simulated data of total Ada (*bottom*) are shown. The cell-average data from >100 cells is shown in gray. The arrow in (*C*) indicates the time when 200 *μ*M MMS was removed. To see this figure in color, go online.

I simulated time traces of Ada expression in single cells using a custom implementation of Gillespie’s algorithm in MATLAB (45). To this end, the Eqs. 1, 2, and 3 of the deterministic model were expressed as elementary unimolecular or bimolecular reactions. Gillespie’s algorithm assumes memoryless kinetics, which is appropriate for transitions between discrete chemical states in which the system is defined entirely by its present state (Markov process). Stochasticity arises because of the discreteness of the states of the system (i.e., the integer number of molecules in the cell) with spontaneous random transitions given by the elementary reactions of the system. At a given time point, the waiting time until the next transition is drawn from an exponential distribution with an expectation value given by the inverse of the sum of all rates exiting that state (i.e., the rates of molecule production and conversion). Which of the possible transitions occurs is then chosen randomly with probabilities according to the relative rates of the reactions. Initial molecule numbers were drawn from a Poisson distribution defined by the basal expression rate (see Fig. 2 *A*). Cell growth was modeled as a deterministic exponential increase in cell volume over the course of a generation. The promoter association rate *k*_on_ was scaled by the cell volume at each time point. The other rates were approximated to be independent of cell volume. At the end of a generation, the cell volume was halved, and the number of remaining molecules was randomly drawn from binomial distributions to account for stochastic partitioning of molecules with equal probability at cell division. Molecule numbers were divided by the cell volume at each time point as for experimental data.

### Model parameters

Parameters were either obtained by direct experimental measurement (18) or by matching the model output to experimental observations (Table 1). One set of parameters was used for all the deterministic or stochastic model realizations in this article. The cell generation time of 42 min in supplemented M9 glucose medium (or 75 min in M9 glycerol, Fig. 5) was obtained directly by timing cell division events in the time-lapse microfluidics data. Fluorescence intensities were calculated from the average pixel intensities within the segmented cell areas. To correct for the background fluorescence, the intensity before MMS treatment was subtracted on a per-cell basis. To directly compare Ada abundances between experiment and model, fluorescence intensity units were converted to molecule concentrations, as described previously (18, 46). The detection efficiency of fluorescent Ada-mYPet was estimated to account for incomplete fluorescent protein maturation. Considering a maturation half time of 9.7 min for mYPet (47) at 37°C and a cell generation time of 42 min, fluorescent molecules represent a fraction 1/(1 + 9.7 min/42 min) = 80% of the total Ada abundance.

**Table 1:** Parameter Values Used for All Plots in This Article

Parameter	Value
*k*_basal_	1.25 molecules generation^−1^
*k*_ind_	1250 molecules generation^−1^
*k*_on_	10 generation^−1^
*k*_off_	1200 molecules generation^−1^
*k*_me_	1.25 molecules [MMS] ^−1^ generation^−1^
*ρ*	0.65 generation^−1^

MMS concentration in mM.

## Results

### A quantitative model of the adaptive response

I first examined whether the proposed model (Fig. 1 *A*) could reproduce the cell-average steady-state expression of Ada after continuous treatment with MMS for 20 cell generations. Experiments showed that Ada expression was very low in the absence of damage treatment and for concentrations below a threshold of 200 *μ*M MMS. A switch-like dose response occurred for concentrations above 350 *μ*M MMS in which Ada abundance saturated with a very narrow transition region of intermediate Ada expression levels. The analytical steady-state solution of the model accurately reproduced the dose response curve (Fig. 1 *B*). I also tested a more complex version of the model in which methylation of Ada at both Cys38 and Cys321 is required for *ada* gene induction (27, 48). Examining the dose response for a range of methylation rates showed that a good match to experimental data could only be obtained when methylation of one of the cysteines is fast compared to the other (Fig. S1). This indicates either that single methylation is sufficient for *ada* induction or that one of the methylation reactions is rate limiting in vivo, justifying the use of a single effective methylation rate in the model.

Imaging cells inside a microfluidic device allowed following the gene expression dynamics of the adaptive response in real time (18). Ada-mYPet fluorescence was used to measure the total Ada abundance (unmethylated Ada and meAda) per cell using time-lapse imaging for hundreds of cells over tens of generations per experiment. Averaging the measured fluorescence signal of all cells at each time point showed that continuous treatment with high MMS concentrations (>350 *μ*M) caused rapid activation of Ada expression within two cell generations, and steady-state expression was reached within ∼10 generations (Fig. 1 *C*). For lower MMS concentrations (<350 *μ*M), initial response activation was delayed by more than five generations, and expression reached steady state only after ∼20 generations of treatment. The numerical solution of the model closely matched the measured dynamics (Fig. 1 *C*), using the same set of parameters as for the steady-state analysis. Furthermore, the model confirms that the removal of MMS leads to deactivation of the adaptive response by the dilution of Ada and meAda molecules as a result of cell division (Fig. 1 *D*). The abundance of meAda decayed exponentially immediately after MMS removal, whereas Ada expression remained induced for several generations until meAda levels had diminished. This deactivation delay scaled with the concentration of MMS that cells were exposed to previously (Fig. S2). Consequently, a cell’s memory of the stress and adaptation are prolonged after severe damage exposure. There were no signs of hysteresis effects after MMS removal, as expected for the noncooperative promoter binding of meAda (42).

### Poisson fluctuations of Ada expression in the absence of DNA damage

Stochastic effects appear to play a key role in the dynamics of the adaptive response at the single-cell level. Single-molecule counting of Ada-mYPet showed that the average production rate in the absence of DNA methylation damage is as low as one Ada molecule per cell generation (18). This is equivalent to a population mean of 1.4 Ada molecules per cell, given that the average loss rate by cell division is ln (2) per cell generation. The distribution of Ada numbers ranged from 0 to ∼6 molecules per cell. Spontaneous induction of higher Ada expression in the absence of MMS treatment was never observed in experiments (Fig. 2 *A*, inset). Ada copy numbers were well described by a simulated Poisson distribution when the mean was fixed by the average expression from experiments (Fig. 2 *A*). The integer numbers of Ada molecules can be viewed as discrete cell states, and transitions between these states occur with a constant (memoryless) probability given by the average production and loss rates.

Many genes are expressed in bursts, in which multiple mRNAs are produced in a short interval, and each transcript is translated repeatedly (49), which broadens protein expression distributions (50). The close fit of the Poisson distribution demonstrates a lack of expression bursting for Ada, which can be explained by the low translation efficiency and short half life of *ada* mRNAs (43, 44). It has also been shown that periodic changes in the gene copy number due to DNA replication result in gene expression variation (51, 52). However, because the *ada* gene is located close to the chromosome terminus region, the gene is present at a single gene copy until late in replication and therefore expected to show little expression variation over the cell cycle.

### A stochastic model recapitulates single-cell response dynamics after DNA damage treatment

In contrast to the gradual response induction suggested by the numerical solution of the model (Fig. 1 *D*), single-cell time-lapse imaging revealed significant heterogeneity in *ada* expression after MMS exposure (18). Continuous treatment with low concentrations of MMS (50–100 *μ*M) caused stochastic pulses of Ada expression but did not sustain the conversion of Ada to the meAda transcription activator (Fig. 2 *B*). Because the pulses are rare, the cell-average expression is close to the low average value predicted by the deterministic version of the model (gray curves in Fig. 2, *B*–*D*). Intermediate MMS concentrations (200–350 *μ*M) resulted in a persistent Ada induction once the response was activated, but activation times were extremely broadly distributed across cells (Fig. 2 *C*). Delays of more than 20 generations were frequently observed, a time in which a single cell can grow into a colony of millions. Therefore, the slow induction of Ada expression seen in the bulk-average curves (Fig. 1 *C*) is caused by a gradual increase in the proportion of induced cells over time. Even at high MMS concentrations (>500 *μ*M), activation times differed by multiple generations between cells (Fig. 2 *D*). Contrary to response activation, removal of MMS caused all cells to switch off the adaptive response uniformly (Fig. 2 *C*). Residual heterogeneity in the response deactivation times between cells was similar for different MMS concentrations, consistent with the simple model of deactivation by dilution (Fig. S2).

I tested whether the proposed model could explain aspects of the observed cell-to-cell variation. Importantly, the microfluidic imaging system ensures that cells grow under constant identical conditions, such that any heterogeneity can be attributed to stochastic processes intrinsic to each cell. I hypothesized that incorporating the discrete nature of molecule numbers and probabilistic reaction kinetics into the model could account for the stochastic response dynamics. This hypothesis was driven by the fact that Poisson fluctuations are especially pronounced for low numbers of molecules as measured for Ada. Moreover, the positive feedback loop of Ada can amplify any initial fluctuations (53).

Stochastic simulations provide a general approach for generating single-cell trajectories that can be directly compared to experimental data (54). To this end, I expressed the model equations as unimolecular or bimolecular elementary reactions and used Gillespie’s algorithm (45) to create probabilistically exact realizations of the proposed model. I used the same parameter values as for the deterministic model. Remarkably, simulated trajectories closely resembled the complex dynamics of the adaptive response in single cells over the whole range of MMS concentrations used in the experiments (Fig. 2). In particular, simulations reproduced the random Ada expression bursts at low MMS as well as the stochastic activation followed by sustained Ada expression at high MMS concentrations. Simulated cell traces also showed uniform deactivation of Ada expression after MMS removal. Importantly, no additional features or noise terms had to be added to the model to achieve these features.

### Poisson noise in basal Ada expression dictates stochastic response delays

**Figure 3:**
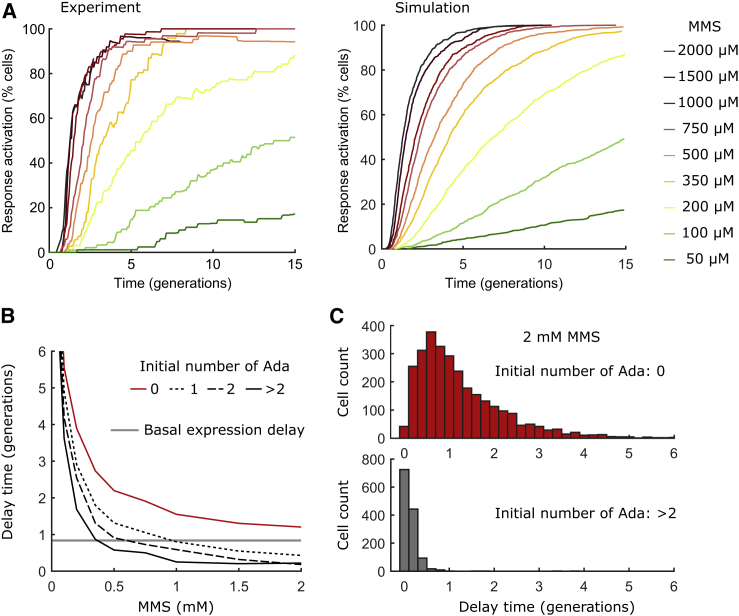
Gene expression noise randomizes the activation of the adaptive response. (*A*) Response activation time distributions are shown. Cumulative distributions show the percentage of cells that activated the adaptive response after MMS exposure. The activation time corresponds to the time when Ada levels crossed a threshold of 23 molecules per cell (a threshold close to the experimental detection limit). The experimentally measured distributions from time-lapse microscopy include between 59 and 149 cells for each MMS concentration (*left*). Model results are from 1000 independent Gillespie simulations for each MMS concentration (*right*). Identical data analysis was performed for simulated and experimental cell trajectories. (*B*) Shown is the average delay time between MMS addition and the generation of the first meAda molecule from 1000 simulated trajectories, conditional on the initial number of Ada molecules at the time of MMS addition. The red curve is for cells that initially had zero Ada molecules, the dotted black line is for one Ada molecule, the dashed black line is for two Ada molecules, and the solid black line is for more than two Ada molecules. The average waiting time between basal expression events is shown in gray. (*C*) Shown is the simulated distribution of response delay times after 2 mM MMS treatment for cells with initially zero Ada molecules (*red*) or more than two Ada molecules (*gray*). To see this figure in color, go online.

For a quantitative comparison of experiments and model simulations, I evaluated the distribution of delay times between the addition of MMS and the first activation of the adaptive response in single cells (Fig. 3 *A*). The delay time distributions from stochastic simulations of the model closely resembled those from experiments. However, it is evident that the fluctuations in Ada expression after response activation are larger in experiments than in the simulated trajectories (Fig. 2). The additional variation likely reflects “extrinsic noise” (55) due to fluctuations in factors that influence Ada expression but were not included in the model, such as RNA polymerase and ribosome concentrations and variation in the length of the cell cycle. Nevertheless, the close match of the simulated and experimental delay time distributions shows that stochasticity in the initial activation time of the response is not influenced by such external noise sources but can be solely attributed to basic Poisson fluctuations in *ada* gene expression.

In particular, noise in the low basal expression of Ada is responsible for a subpopulation of 20–30% of cells that do not contain any Ada molecules (18). These cells are thus unable to activate the autoregulatory adaptive response until they produce at least one Ada molecule. For simulated data, it is possible to calculate response delay times conditional on the initial number of Ada molecules at the time of MMS exposure. This analysis confirmed that the average delay time between MMS addition and the production of the first meAda molecule converges to zero with increasing MMS concentration only for cells that initially contain one or more Ada molecules (Fig. 3, *B* and *C*). But for cells lacking any Ada molecules, the average delay time approaches a limit defined by the average waiting time between stochastic basal expression events (Fig. 3, *B* and *C*). In the model, the basal *ada* production is a zero-order reaction with an MMS-independent rate constant. Thus, response activation for cells without Ada molecules follows a memoryless process with an exponential distribution of delay times (Fig. 3 *C*), as seen in experiments (18).

### Testing the predictive power of the model using experimental perturbations

**Figure 4:**
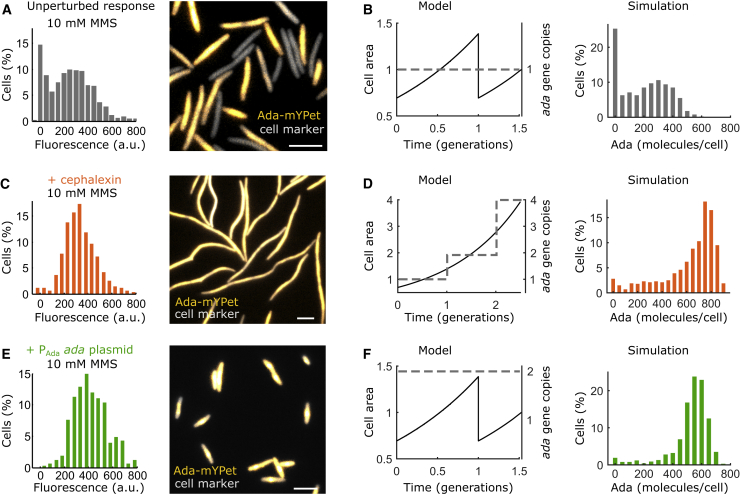
Experimental perturbations to test the model of the adaptive response. The distributions of total Ada abundance per cell were generated from 1000 simulation repeats. Experimental data are from microscopy snapshots with Ada expression shown in fluorescence units (a.u.), whereas simulation outputs are in molecule numbers. Scale bars, 5 *μ*m. (*A*) Unperturbed Ada-mYPet expression after treatment with 10 mM MMS for 1 h is shown. (*B*) Simulation of the unperturbed Ada response according to the experiment in (*A*) is shown. (*C*) Shown is the inhibition of cell division by cephalexin treatment for 45 min, followed by 10 mM MMS treatment for 1 h before imaging. (*D*) Shown is the simulation of the experiment in (*C*). Lack of cell division abolishes partitioning loss of molecules. Cell area and *ada* gene copy numbers increase because of continued cell growth and replication. (*E*) Shown is a mild overexpression of Ada by transforming cells that express endogenous Ada-mYPet with a very low copy number MiniF plasmid (∼1–2 per cell) that expresses unlabeled Ada from the P_Ada_ promoter. Cells were treated with 10 mM MMS for 1 h before imaging. (*F*) Shown is the simulation of the experiment in (*E*) by duplicating the *ada* gene. The number of molecules expressed from one of the genes is shown (corresponding to the labeled *ada-mypet* in the experiment). To see this figure in color, go online.

The predictive power of the model was tested by comparison to experiments in which cells were subjected to perturbations that alter the regulation of the adaptive response in a defined manner (18) (Fig. 4, *A* and *B*). When cell division was inhibited for 45 min using the antibiotic cephalexin before MMS treatment, Ada molecules accumulate in filamentous cells, and the activation of the adaptive response becomes uniform in the population (18) (Fig. 4 *C*). In the model, cell growth without division resulted in an exponential increase of cell area and an amplification of *ada* gene copies over time. This, together with the lack of molecule partitioning, was sufficient to generate a uniform response in the simulations (Fig. 4 *D*). As an alternative perturbation to reduce the number of cells with zero Ada molecules, endogenous Ada-mYPet expression was supplemented with a plasmid that is present at one to two copies per cell and expresses *ada* from the P_Ada_ promoter. The slight overexpression of Ada strongly reduced cell-to-cell variation upon MMS treatment and eliminated the population of cells with a delayed response in experiments (18). I modeled this perturbation by duplicating the *ada* gene in the simulations (Fig. 4 *C*). This alteration resulted in uniform response activation as seen in experiments (Fig. 4 *C*). However, the simulations generated higher Ada expression levels than measured experimentally, likely because Ada overexpression is toxic in experiments (18).

### Effect of cell growth rate on the response strength

**Figure 5:**
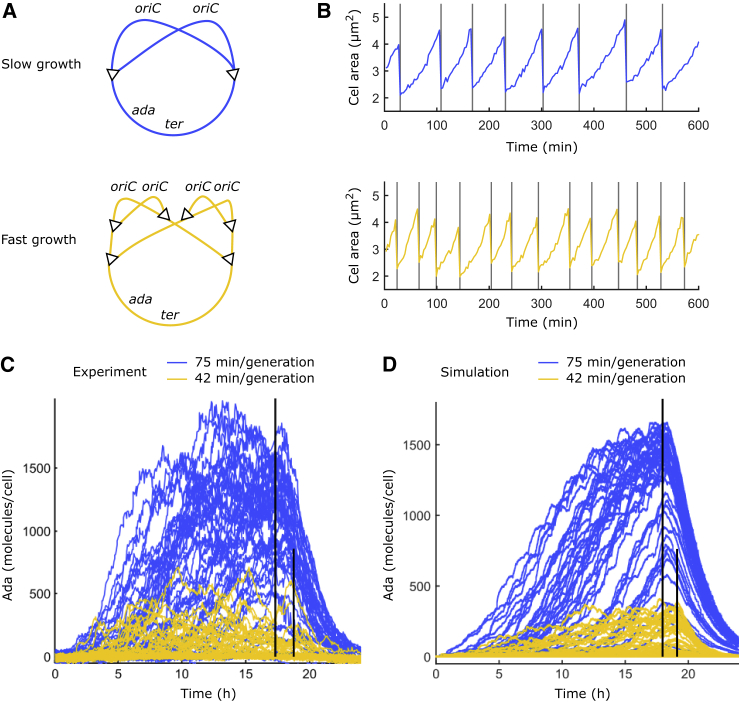
Effect of cell growth rate on the strength of the adaptive response. (*A*) Schematic shows replication of the circular *E. coli* chromosome during slow (*blue*) and fast (*yellow*) cell growth. DNA replication initiates at *oriC* and proceeds bidirectional toward *ter*. For fast growth, initiation occurs before completion of the previous replication round. The *ada* gene is located close to *ter*, so the gene copy number does not increase at faster growth. (*B*) Example time traces show the size of a cell growing in minimal medium plus glycerol at 75 min per generation (*blue*) or in minimal medium plus glucose at 42 min per generation (*yellow*). Division times are indicated by vertical lines. (*C*) Single-cell time traces of Ada expression from time-lapse microscopy with 100 *μ*M MMS treatment for cells growing in glycerol (*blue*) or glucose (*yellow*) medium, respectively. Vertical lines indicate times when MMS was removed. (*D*) Simulation of the experiment in (*B*) is shown. Single-cell traces of total Ada for slow (*blue*) and fast (*yellow*) growth with 100 *μ*M MMS treatment are shown. To see this figure in color, go online.

The doubling time of *E. coli* in rich growth medium is shorter than the time required to replicate the chromosome. Cells achieve this by initiating new rounds of replication before completion of the previous round (56). The early duplication of genes close to the replication origin increases their expression proportional to the replication initiation frequency and thus counteracts the dilution of these proteins at faster growth rates. The abundance of Ada, however, being expressed from the *ada* gene at 49.7 min on the chromosome map in the vicinity of the terminus region, is expected to drop with increasing growth rates (Fig. 5 *A*). I tested this prediction experimentally by growing cells in minimal medium supplemented with glucose or glycerol carbon sources, which resulted in generation times of 42 min or 75 min, respectively (Fig. 5 *B*). Indeed, a strong Ada response occurred when slowly growing cells were treated with 100 *μ*M MMS, whereas faster growth did not sustain a response at the same MMS concentration (Fig. 5 *C*). This prediction can be tested by the model of the Ada response. I fixed the Ada expression rate and gene copy number while modifying the time between division events, hence the molecule dilution rate. Simulations confirmed the inverse relation between the growth rate and the strength of the adaptive response (Fig. 5 *D*). This finding raises the question whether natural fluctuations in growth rates also modulate the adaptive response in a similar way. In the microfluidic chip, cell generation times varied over time and between cells with a SD of ±8 min per generation in M9 glucose medium. Indeed, slower growth correlated with elevated Ada expression in single cells (Fig. S3).

## Discussion

The role of noise in the fidelity of DNA repair has been investigated in eukaryotes, in which nucleotide excision repair involves stochastic and reversible assembly of repair factors into large complexes (57, 58). Collective rate control renders the overall repair pathway robust to variation in the abundances of the individual components (39). The situation is opposite for damage signaling by Ada, which acts alone in the regulation of the adaptive response and feedback amplification results in extreme sensitivity to gene expression noise. Remarkably, it has been shown that random variation in the abundance of Ada by just a single molecule is responsible for separating isogenic cells into distinct populations that either induced or failed to induce the DNA damage response. This has important consequences because the lack of a damage response decreases survival and increases mutation in those cells (17).

The adaptive response has been described as “a simple regulon with complex features” (27). Instead of attempting to incorporate all mechanistic details, the model described in this article attempts to reduce the *ada* regulation to its central features. For example, methylation of both Cys38 and Cys321 residues in the N- and C-terminal Ada domains is required for optimal activation of the P_Ada_ promoter (48). I found that a single effective methylation rate is sufficient to reproduce experimental observations without the need to distinguish between single or double methylation of Ada. Another abstraction is that the amount of DNA damage was not explicitly included in the model but rather absorbed in a constant methylation rate that is proportional to the MMS concentration. Nevertheless, it is expected that methylation lesions initially accumulate during the adaptive response delay. Subsequently, as Ada repair activity increases, the number of lesions will decrease, effectively generating a negative feedback loop. Although such a combination of positive and negative feedback mechanisms could generate a pulse of Ada expression (53), this has not been observed in experiments. To test this scenario with a model that incorporates DNA damage as a variable, future studies could directly quantify the number of lesions and the repair activity of Ada in vivo, as previously shown for other enzymes involved in DNA alkylation repair (7). It is also worth considering that the adaptive response interacts with other cellular responses and processes. For instance, the alternative *σ* factor RpoS induces Ada expression upon entry into the stationary phase (27), whereas the SOS response is crucial for initial survival of alkylation damage and contributes to alkylation-induced mutagenesis (17). Considering these simplifications, it is remarkable that the most parsimonious model of the adaptive response not only succeeds in quantitatively explaining a large spectrum of stochastic single-cell dynamics but also in predicting the system’s behavior after different experimental perturbations.

## Supporting Material

Document S1. Figs. S1–S3

Document S2. Article plus Supporting Material
