# Supplementary material for: A Quantitative Model Explains Single-Cell Dynamics of the Adaptive Response in Escherichia coli
Source: Biophys J. 2019 Aug 15;117(6):1156–65. doi: 10.1016/j.bpj.2019.08.009 (PMC6818145; doi:10.1016/j.bpj.2019.08.009)
Supplement: Document S1. Figs. S1–S3 [file mmc1.pdf]

**Biophysical Journal, Volume *117***

## Supplemental Information

# A Quantitative Model Explains Single-Cell Dynamics of the Adaptive Response in *Escherichia coli*

**Stephan Uphoff**

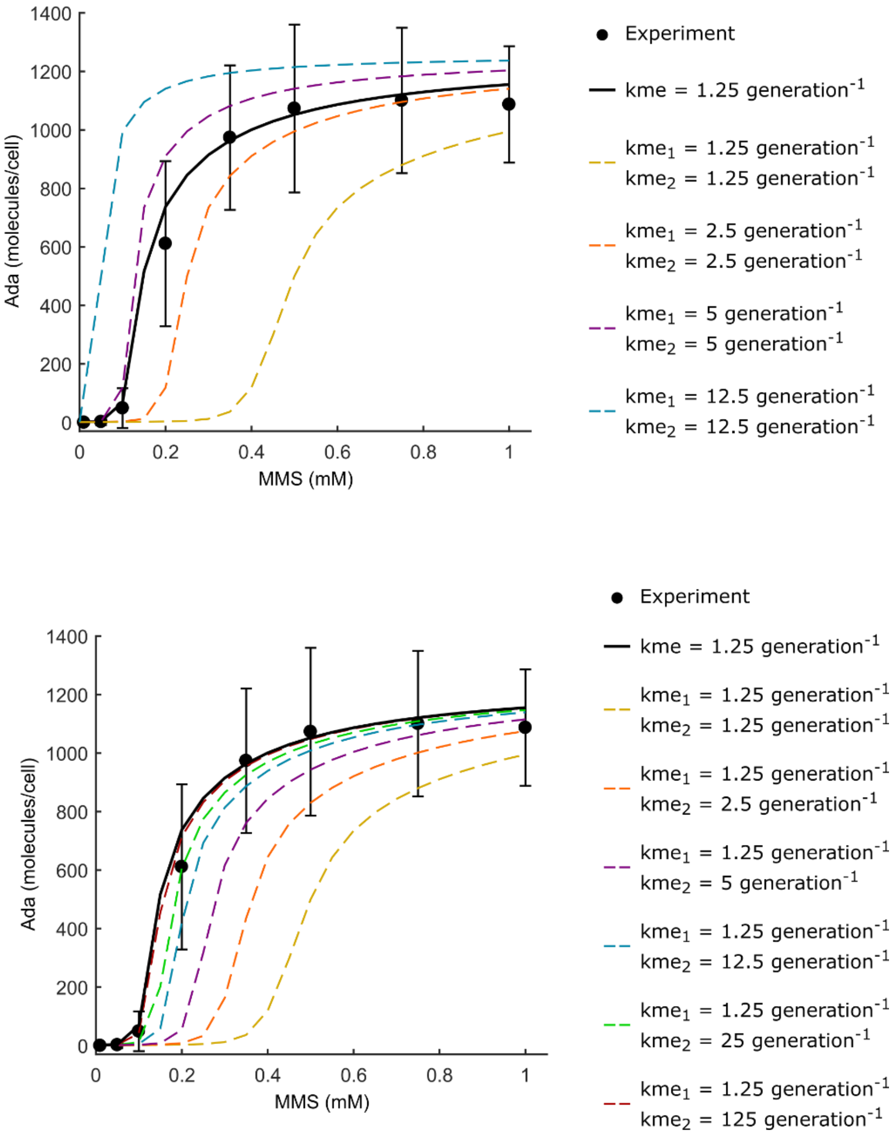


**Figure S1 Model incorporating double methylation of Ada.** Experimental data show cell-average steady-state expression of Ada after constant treatment with different doses of MMS for 20 cell generations (mean ± standard deviation). Curves correspond to numerical model solutions for the total Ada abundance. Dashed lines show the dose response curve for a model in which two independent methylation reactions of Ada (i.e. at residues Cys38 and Cys321) are required for ada gene induction. Colours indicate different combinations of reaction rates for the two methylation sites. The double methylation model reproduces experimental data only when one of the methylation rates is high compared to the other. This justifies the use of a model with a single effective methylation rate (solid black line).

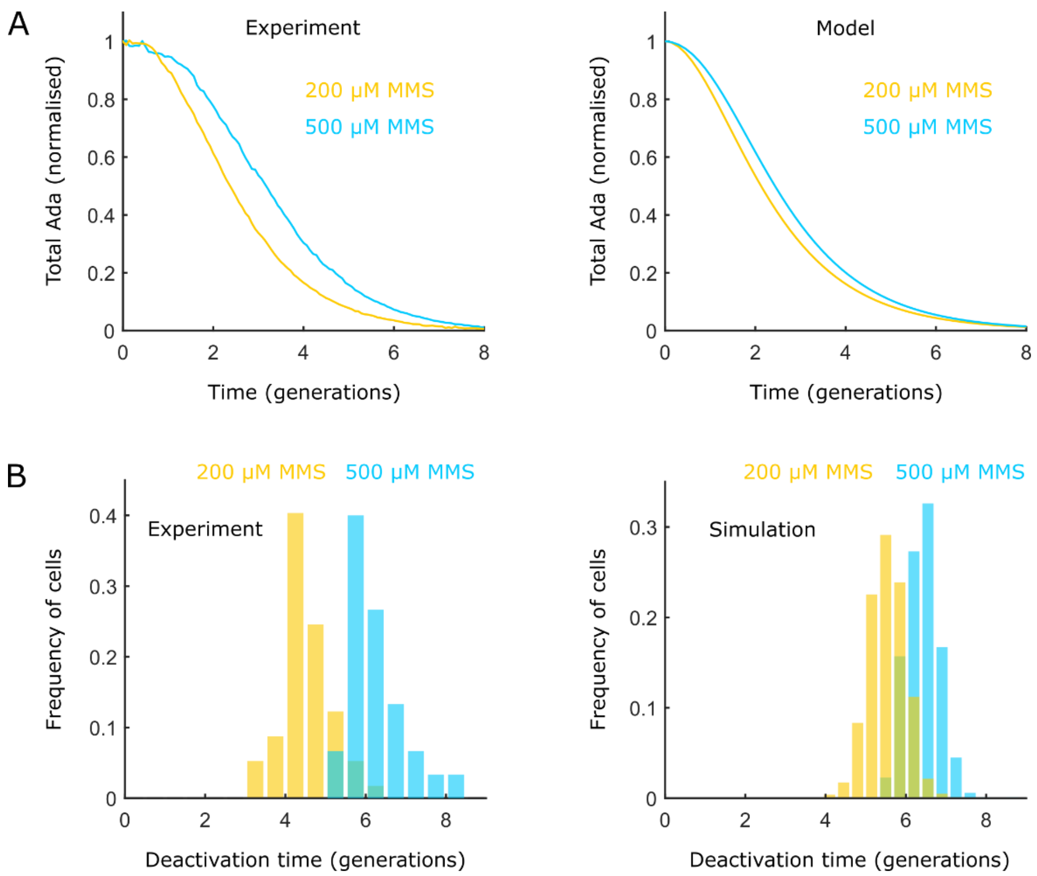


**Figure S2 Ada response deactivation after exposure to different MMS concentrations. (A)** Experimental data and numerical solution of the model show the cell-average total Ada abundance. Curves were normalised to the Ada abundance at time 0 when MMS was removed. The time delay before the Ada abundance decays is prolonged at increased MMS concentration. **(B)** Distribution of Ada response deactivation times for single cells from experimental data and simulations after exposure to 200 µM and 500 µM MMS, respectively. The deactivation time was measured as the time post MMS removal when total Ada passed below a threshold of 50 molecules/cell.

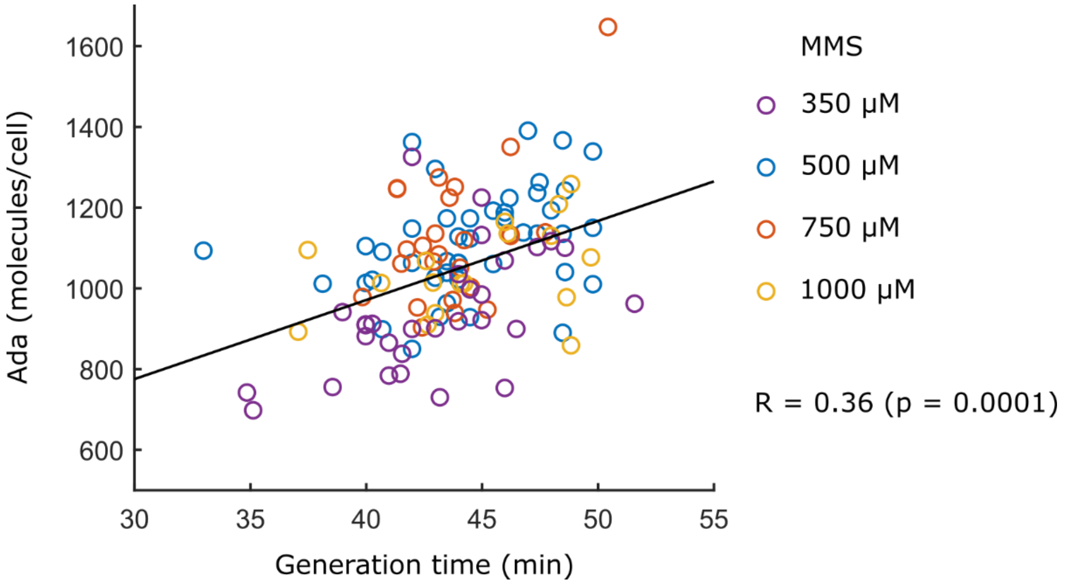


**Figure S3 Correlation between Ada abundance and cell generation time.** Experimental data show time-averaged Ada abundance after response induction plotted versus the average generation time of single cells in experiments during treatment with different MMS concentrations. Line: linear fit. R: Pearson correlation coefficient.
